# Efficacy and Safety of Intravenous Thrombolysis with Tenecteplase in Patients with Wake-Up Branch Atheromatous Disease

**DOI:** 10.1007/s12975-026-01443-8

**Published:** 2026-05-01

**Authors:** Lili Zhu, Ying Zhang, Jiarong Li, Liang Song, Haoran Li, Yang Yang, Lijuan Liu, Shaolei Zhang, Xiaofei Gao, Weifeng Chen, Guo Rui, Haiqiang Qin, Mingbo Tian, Shengqi Fu

**Affiliations:** 1 Department of Neurology, The Fifth Clinical Medical College of Henan, University of Chinese Medicine (Zhengzhou People’s Hospital), No. 33 Huanghe Road, Jinshui District, Zhengzhou, Henan Province 450003 China; 2 Department of Neurology, Hopeshine–Minsheng Hospital of Xinzheng, No. 126, Jiefang North Road, Xinzheng City, Zhengzhou, 451100 China; 3 Department of Neurology, People’s Hospital of Zhongmu, Zhongmu County, No. 64, Jiefang Road, Chengguan, Zhongmu, 451450 China; 4 Department of Neurology, Zhengzhou Zhongkang Hospital, No. 298, Nongye Road, Xinmi City, Zhengzhou, 450007 China; 5 Department of Neurology, Dengfeng People’s Hospital, No. 2, Zhongyue Street, Dengfeng, 452470 China; 6 Department of Neurology, Xingyang People’s Hospital, No. 22, Suohe West Road, Xingyang, 450100 China; 7 Department of Neurology, Zhengzhou Hospital of Traditional Chinese Medicine, No. 65, Cultural Palace Road, Zhengzhou, 450007 China; 8 Department of Neurology, Beijing Tiantan Hospital, Capital Medical University, No. 119, South 4th Ring West Road, Fengtai District, Beijing, 100070 China; 9 Office of Research Administration, The Fifth Clinical Medical College of Henan, University of Chinese Medicine (Zhengzhou People’s Hospital), No. 33 Huanghe Road, Jinshui District, Zhengzhou, 450003 China

**Keywords:** Branch atheromatous disease, DWI/FLAIR mismatch, Early neurological deterioration, Intravenous thrombolysis, Tenecteplase, Wake-up stroke

## Abstract

Branch atheromatous disease (BAD), a subtype of acute ischemic stroke (AIS), is associated with a high risk of early neurological deterioration (END) and poor prognosis. Wake-up stroke (WUS), comprising 20%–30% of AIS cases, is typically excluded from thrombolysis because of unknown onset time. Using diffusion-weighted imaging/fluid-attenuated inversion recovery (DWI/FLAIR) mismatch, we evaluated the efficacy and safety of Tenecteplase (TNK) thrombolysis in patients with BAD-related WUS. We retrospectively recruited 1,062 patients from seven Zhengzhou hospitals between January 2021 and June 2025. The patients received either TNK (n = 338) or dual antiplatelet therapy (n = 724). Propensity score matching (PSM; 1:1, caliper 0.02) yielded 292 matched pairs. Early neurological changes were evaluated using the National Institutes of Health Stroke Scale (NIHSS), and 90-day outcomes were evaluated using the modified Rankin Scale (mRS). After PSM, TNK significantly reduced the risk of END (odds ratio [OR] = 0.425, 95% confidence interval [CI]: 0.262–0.689; P < 0.001). Patients treated with TNK were more likely to achieve good functional outcomes (modified Rankin Scale [mRS] 0–1 and 0–2) with fewer poor outcomes (mRS ≥ 4). There were no significant differences in symptomatic intracranial hemorrhage, other bleeding events, or mortality among the groups. DWI/FLAIR mismatch-guided TNK thrombolysis appears to overcome the limitations of an unknown onset time in WUS and may counteract the progressive pathophysiology of BAD. TNK intravenous thrombolysis may be a safe and effective treatment for patients with wake-up BAD and DWI/FLAIR mismatches.

## Introduction

Branch atheromatous disease (BAD) is a subtype of acute ischemic stroke (AIS). As atherosclerosis-induced stenosis and occlusion of perforating arteries (approximately 700–800 μm in diameter) occur at the origin of the parent artery, symptoms in the acute phase tend to fluctuate repeatedly, with a high incidence of early neurological deterioration (END) and low clinical prognosis [1]. Intravenous thrombolysis is the primary method for restoring cerebral perfusion and reducing the risk of END in acute AIS [2, 3]. A multicenter study showed that intravenous injection of Tenecteplase (TNK; 0.25 mg/kg), a novel thrombolytic drug, was non-inferior to alteplase in terms of efficacy and safety endpoints in eligible patients with AIS, providing strong evidence for its clinical use [4, 5].

Wake-up stroke (WUS) accounts for 20%–30% of AIS cases; however, there is no significant difference in clinical and imaging characteristics between patients with and without WUS. Although the END and disability rates are high, WUS has traditionally been excluded from thrombolysis because the onset time remains unknown, resulting in unmet treatment needs [6]. The mismatch between diffusion-weighted imaging (DWI) and fluid-attenuated inversion recovery (FLAIR) provides a new evaluation tool for thrombolytic therapy in WUS. Thomalla et al. demonstrated that DWI/FLAIR-guided WUS thrombolysis could improve 90-day prognoses without increasing the risk of bleeding, thereby overcoming time window limitations [7].

However, TNK efficacy in the general AIS population and advances in DWI/FLAIR-guided WUS thrombolysis have not addressed the special subpopulation of patients with post-waking BAD. The pathological mechanism of BAD is distinct from that of other AIS subtypes, and the uncertainty of the onset time in WUS increases treatment difficulty. Currently, clinical evidence on the efficacy and safety of TNK thrombolysis for BAD in patients with WUS with DWI/FLAIR mismatch is lacking. Therefore, this study aimed to evaluate the efficacy and safety of TNK intravenous thrombolysis in patients with wake-up BAD presenting with DWI/FLAIR mismatch. We hypothesized that in post-awakening patients with BAD who exhibit DWI/FLAIR mismatch, TNK thrombolytic therapy would demonstrate superior efficacy and safety compared with the expected outcomes without thrombolytic treatment, thereby providing evidence-based support for acute management and optimization of diagnostic and treatment strategies.

## Methods

### Ethical Approval

 This multicenter retrospective case–control study was approved by the Ethics Committee of the Fifth Clinical Medical College of Henan University of Chinese Medicine, which is affiliated with Zhengzhou People’s Hospital (approval number 2017L01098). All procedures were performed in compliance with the guidelines outlined in the Declaration of Helsinki. Given the retrospective design of this study, the requirement for written informed consent was waived.

### Study Participants

**Fig. 1:**
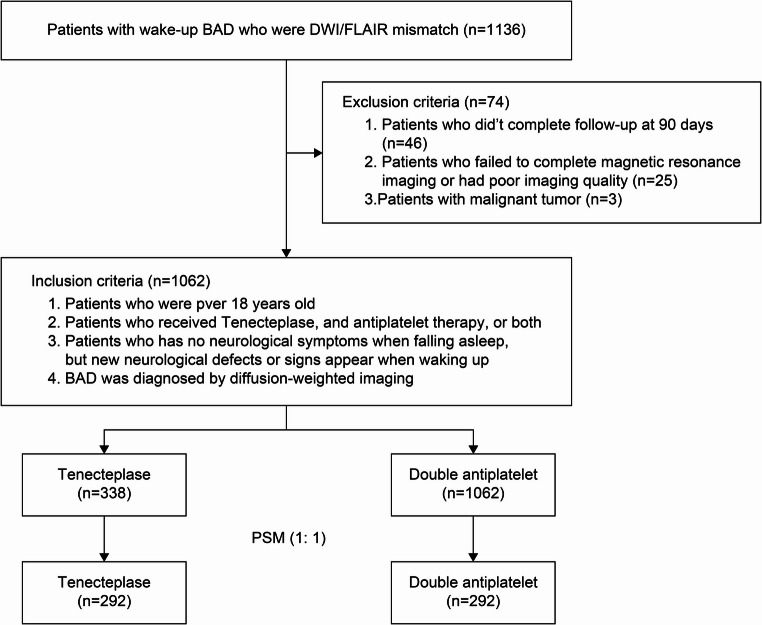
Flowchart of eligible participant selection. BAD, branch atheromatous disease

Clinical data were collected from patients diagnosed with BAD who were admitted to the Zhengzhou People’s Hospital, Hopeshine-Minsheng Hospital of Xinzheng, People’s Hospital of Zhongmou, Zhengzhou Zhongkang Hospital, Dengfeng People’s Hospital, Xingyang People’s Hospital, or Zhengzhou Hospital of Traditional Chinese Medicine between January 2021 and June 2025. The inclusion criteria were: (1) meeting the diagnostic criteria specified in the Chinese expert consensus on BAD for lenticulostriate artery territory ischemic stroke (DWI must show that the infarct lesions in the corresponding blood supply area involve ≥3 levels) or for paramedian pontine territory ischemic stroke (DWI must demonstrate continuity between the infarct and the ventral surface of the brainstem, with the lesion located in the midline-adjacent region yet confined to a single side); (2) age >18 years; (3) no obvious abnormal findings during sleep, with new-onset neurological deficit symptoms and signs appearing after awakening; (4) completion of cranial computed tomography (CT), magnetic resonance imaging (MRI), and magnetic resonance angiography (MRA) examinations before treatment; (5) presence of DWI/FLAIR mismatch, defined as a hyperintense area on DWI with no corresponding hyperintensity on FLAIR; and (6) availability of complete 90-day follow-up data. The exclusion criteria were: (1) a history of intracranial hemorrhage within the previous 3 months; (2) cranial MRI showing large-vessel disease or intracranial vascular stenosis >50%; (3) comorbidities with severe hepatic/renal dysfunction, severe heart failure, or coagulation disorders; and (4) previous treatment with other intravenous thrombolytic drugs. A flowchart illustrating the selection process for eligible study participants is shown in Figure 1.

### Data Collection

 Data extracted from patients encompassed demographic information (age and sex), vascular risk factors (hypertension, diabetes mellitus, hyperlipidemia, atrial fibrillation, prior transient ischemic attack/stroke, smoking, and alcohol consumption), baseline measurements (blood pressure, blood glucose, body mass index [BMI], NIHSS score, and Barthel Index [BI] score), time from the midpoint of total sleep to medication administration, and baseline laboratory parameters (total cholesterol, triglycerides, low-density lipoprotein cholesterol, high-density lipoprotein cholesterol, homocysteine, C-reactive protein, and D-dimer).

### Therapeutic Approach

Treatment allocation was determined through standardized clinical decision-making, guided by institutional stroke management protocols and individualized patient characteristics. Patients were assigned to receive TNK thrombolysis if they fulfilled all the prespecified inclusion criteria: presence of DWI/FLAIR mismatch, absence of thrombolysis contraindications, adherence to the imaging-inferred time window, and provision of verbal informed consent. Conversely, patients were allocated to the DAPT group if they presented with documented thrombolysis contraindications, which included unrecognized bleeding risks, patient refusal to undergo thrombolysis, or delayed presentation exceeding the imaging-inferred time window. In the TNK group (n=338), patients received the recommended 0.25 mg/kg (maximum: 25 mg) of TNK (Guangzhou Mingkang Bioengineering Co., Ltd.; National Medical Product Administration Approval No. S20150001; 1.0×10⁷ IU/16 mg per vial) for intravenous thrombolysis. A cranial CT scan was performed after 24 h to exclude hemorrhage. The patients then received dual antiplatelet therapy (75 mg/day clopidogrel and 100 mg/day aspirin) for 21 days, followed by single antiplatelet therapy (either 75 mg/day clopidogrel or 100 mg/day aspirin). In the dual antiplatelet therapy (DAPT) group (n=724), patients received dual antiplatelet therapy (75 mg/day clopidogrel and 100 mg/day aspirin) within 24 h of symptom onset, continued for 21 days, then followed by single antiplatelet therapy (either 75 mg/day clopidogrel or 100 mg/day aspirin). All antiplatelet therapies were discontinued immediately if cerebral hemorrhage occurred.

### Imaging Assessment

In accordance with the Guidelines for the Early Management of AIS released by the American Heart Association/American Stroke Association (AHA/ASA), all patients underwent MRI within 48 h of admission using a Siemens MAGNETOM 3.0-T Skyra scanner (Erlangen, Germany) fitted with a 12-channel head matrix coil. The scanning sequences and parameters were as follows: T1-weighted imaging (T1WI) with a repetition time (TR) of 1,800 ms and an echo time (TE) of 20 ms; T2-weighted imaging (T2WI) with a TR of 4,700 ms and a TE of 110 ms; T2-weighted FLAIR (T2-FLAIR) with a TR of 8,500 ms and a TE of 150 ms; and DWI with a TR of 4,500 ms, a TE of 82 ms, and b-values of 0 and 1,000 s/mm². During the follow-up period, the NIHSS was used to evaluate neurological functions, such as consciousness level, language capability, visual field, limb strength, and sensation. If a patient presented with new neurological deficit symptoms (e.g., aggravated limb weakness, slurred speech, or impaired consciousness) and their NIHSS score increased by ≥4 points relative to their baseline, this was considered an indicator of potential intracranial hemorrhage. In such instances, CT or MRI was performed promptly to rule out alternative etiologies and validate the clinical diagnosis. Two experienced neurologists conducted DWI/FLAIR mismatch screening and recorded the location of the blood supply area of the cerebral infarction, including the lenticulostriate and pontine middle artery areas.

### Clinical Outcomes and Safety Variables

For efficacy evaluation, the NIHSS score was used to assess END, defined as either an increase of ≥2 points in the total NIHSS score or ≥1 point in the motor subscale within 7 days of admission. Ninety days following symptom onset, the modified Rankin scale (mRS) score was further evaluated through follow-up visits or telephone interviews to determine good prognosis (mRS ≤1), functional independence (mRS ≤2), and poor prognosis (mRS ≥4). For safety assessment, the incidence of symptomatic intracranial hemorrhage (sICH), other bleeding events, and 90-day mortality was compared between the groups based on imaging results and information extracted from medical records.

### Statistical Analysis

All statistical analyses were conducted using GraphPad Prism software (version 9.0; GraphPad Software Inc., San Diego, CA, USA). The Shapiro–Wilk test was applied to assess the normality of continuous variables. Normally distributed data are expressed as x̄±s. Intergroup comparisons were performed using an independent samples t-test. Non-normally distributed data are presented as M (Q1, Q3). Intergroup comparisons were carried out using the Mann–Whitney U test. Categorical variables are presented as n (%). Intergroup comparisons were conducted using the chi-square test, with statistical significance set at P<0.05. Propensity score matching (PSM) was performed between the TNK and DAPT groups at a 1:1 ratio with a caliper value of 0.02. To account for confounders, variables included in the PSM were demographic characteristics (age and sex), risk factors (hypertension, diabetes mellitus, hyperlipidemia, atrial fibrillation, previous transient ischemic attack/stroke, smoking, and alcohol consumption), admission blood pressure, baseline blood glucose, body mass index, baseline NIHSS score, time from the midpoint of total sleep to medication administration, and laboratory indicators (total cholesterol, triglycerides, low-density lipoprotein cholesterol, high-density lipoprotein cholesterol, homocysteine, C-reactive protein, and D-dimer). A binary logistic regression model was used to adjust for baseline variables. TNK treatment was the independent variable, while the primary outcomes (90-day mRS ≤1, END), secondary outcomes (90-day mRS ≤2, 90-day mRS ≥4), and safety outcomes (cerebral hemorrhage, other bleeding events, 90-day mortality) served as dependent variables.

## Results

### Baseline Characteristics

A total of 1,136 patients with WUS who were diagnosed with BAD were retrospectively screened. After applying the eligibility criteria, 74 patients were excluded (Figure 1). Ultimately, 1,062 patients were enrolled in the study, comprising 338 in the TNK group and 724 in the DAPT group. Prior to PSM, significant differences in baseline blood glucose (5.40±1.43 vs. 5.66±1.88), baseline NIHSS scores (4 [3–6] vs. 3 [3–5]), and duration of hospital stay (9 [7–11] vs. 9 [8–12]) were observed between the TNK and DAPT groups, respectively (all P<0.05). No statistically significant differences were noted in the remaining baseline characteristics between the two groups (all P>0.05). After PSM, 292 matched pairs were generated. Although both baseline characteristics were comparable between the two matched cohorts, a significant difference in hospital stay duration persisted after PSM (9 [7–11] vs. 10 [9–12]) (P<0.05; Table1).

**Table 1:** Demographics and clinical characteristics of patients with branch atheromatous disease

Variables	Unmatched cohort				Matched cohort			
	TNK (*n* = 338)	DAPT (*n* = 724)	Statistic	*P*	TNK (*n* = 292)	DAPT (*n* = 292)	Statistic	*P*
Age, mean ± SD, year	62.92 ± 9.62	61.06 ± 10.66	−1.924	0.055	62.27 ± 9.65	61.86 ± 11.28	−0.335	0.738
Male, *n*(%)	186(55.0)	423(58.4)	1.086	0.297	170(58.2)	158(54.1)	0.820	0.365
Risk factors, ***n***(%)								
Hypertension	204(60.4)	417(57.6)	0.722	0.395	114(38.8)	104(35.6)	0.626	0.429
Diabetes	102(30.2)	228(31.5)	0.186	0.666	72(24.7)	82(28.1)	0.791	0.374
Hyperlipidaemia	116(34.3)	218(30.1)	1.894	0.169	130(44.2)	120(41.1)	0.584	0.445
Atrial fibrillation	46(13.6)	94(13.0)	0.079	0.779	36(12.2)	44(15.1)	0.991	0.320
Previous TIA/stroke history	24(7.1)	56(7.7)	0.133	0.715	18(6.1)	22(7.5)	0.459	0.498
Smoking status	74(21.9)	180(24.9)	1.116	0.291	82(27.9)	74(25.3)	0.487	0.485
Alcohol consumption	144(42.6)	316(43.6)	0.102	0.749	122(41.5)	110(37.7)	0.896	0.344
BMI, mean ± SD	24.31 ± 3.23	23.90 ± 3.11	−1.419	0.156	23.96 ± 3.22	24.22 ± 3.11	0.707	0.480
Blood pressure at admission, mean ± SD, mmHg								
SBP	148.38 ± 16.83	147.90 ± 18.14	−0.295	0.768	148.23 ± 16.34	149.55 ± 17.37	0.666	0.506
DBP	87.17 ± 13.83	88.64 ± 12.74	1.206	0.228	88.09 ± 13.86	87.32 ± 12.42	−0.507	0.613
Baseline blood glucose, mean ± SD, mmol/L	5.40 ± 1.43	5.66 ± 1.88	2.264	0.024	5.38 ± 1.69	5.41 ± 1.41	−0.174	0.862
Baseline NIHSS score, median (IQR)	4(3,6)	3(3,5)	−2.885	0.004	4(4,6)	4(3,6)	−0.400	0.689
Baseline BI score, mean ± SD	65.28 ± 14.17	67.67 ± 17.21	1.578	0.115	66.73 ± 14.23	66.12 ± 16.94	−0.335	0.738
SMTM, mean ± SD, h	6.53 ± 1.43	6.77 ± 1.93	1.441	0.150	6.57 ± 1.46	6.61 ± 1.69	−0.206	0.837
Hospital stay, median (IQR), days	9(7,11)	9(8,12)	−2.564	0.010	9(7,11)	10(9,12)	−3.584	< 0.001
Baseline laboratory parameters, mean ± SD, mmol/L								
TC	4.45 ± 1.22	4.50 ± 1.27	0.401	0.689	4.51 ± 1.24	4.59 ± 1.39	0.522	0.602
TG	1.53 ± 0.92	1.58 ± 0.95	0.548	0.584	1.56 ± 0.98	1.61 ± 0.93	0.525	0.600
LDL-C	2.60 ± 0.88	2.55 ± 0.90	−0.699	0.485	2.53 ± 0.84	2.60 ± 0.95	0.661	0.509
HDL-C	1.12 ± 0.29	1.09 ± 0.30	−1.135	0.257	1.09 ± 0.29	1.11 ± 0.32	0.382	0.702
Hcy	16.20 ± 7.29	15.98 ± 6.75	−0.346	0.730	16.02 ± 7.33	15.58 ± 6.62	−0.539	0.590
CRP	2.83 ± 3.28	3.24 ± 6.67	0.764	0.445	2.91 ± 3.39	2.65 ± 2.96	−0.685	0.494
D-dimer	346.66 ± 783.21	382.81 ± 879.34	0.457	0.648	383.88 ± 835.54	399.69 ± 918.70	0.154	0.878

TNK Tenecteplase, DAPT dual antiplatelet therapy, SD standard deviation, IQR interquartile range, NIHSS National Institutes of Health Stroke Scale, SBP systolic blood pressure, DBP diastolic blood pressure, BMI body mass index, LDL-C low-density lipoprotein cholesterol, TG triglyceride, TC total cholesterol, HDL-C high-density lipoprotein cholesterol, TIA transient ischemic attack, Hcy homocysteine, CRP C-reactive protein, SMTM time from midpoint of total sleep to medication administration

 In the unmatched cohort, END occurred in 216 patients (20.3%), with the TNK group exhibiting a significantly lower END rate (14.8%) than the DAPT group (22.9%). After adjusting for confounding variables (P<0.05), univariate logistic regression analysis revealed a statistically significant intergroup difference (adjusted odds ratio [aOR]=0.601, 95% confidence interval [CI]: 0.423–0.853; P=0.004).

 Regarding functional recovery, the TNK group featured a greater proportion of patients achieving mRS scores of ≤1 and ≤2 than the DAPT group. Logistic regression analysis demonstrated a significant association for an mRS ≤1 (aOR=1.587, 95% CI: 1.223–2.060; P<0.001), but not for an mRS ≤2 (aOR=1.241, 95% CI: 0.899–1.712; P=0.190). In contrast, the DAPT group had a significantly higher proportion of patients with mRS scores ≥ 4 (aOR=0.490, 95% CI: 0.283–0.850; P=0.011).

**Fig. 2:**
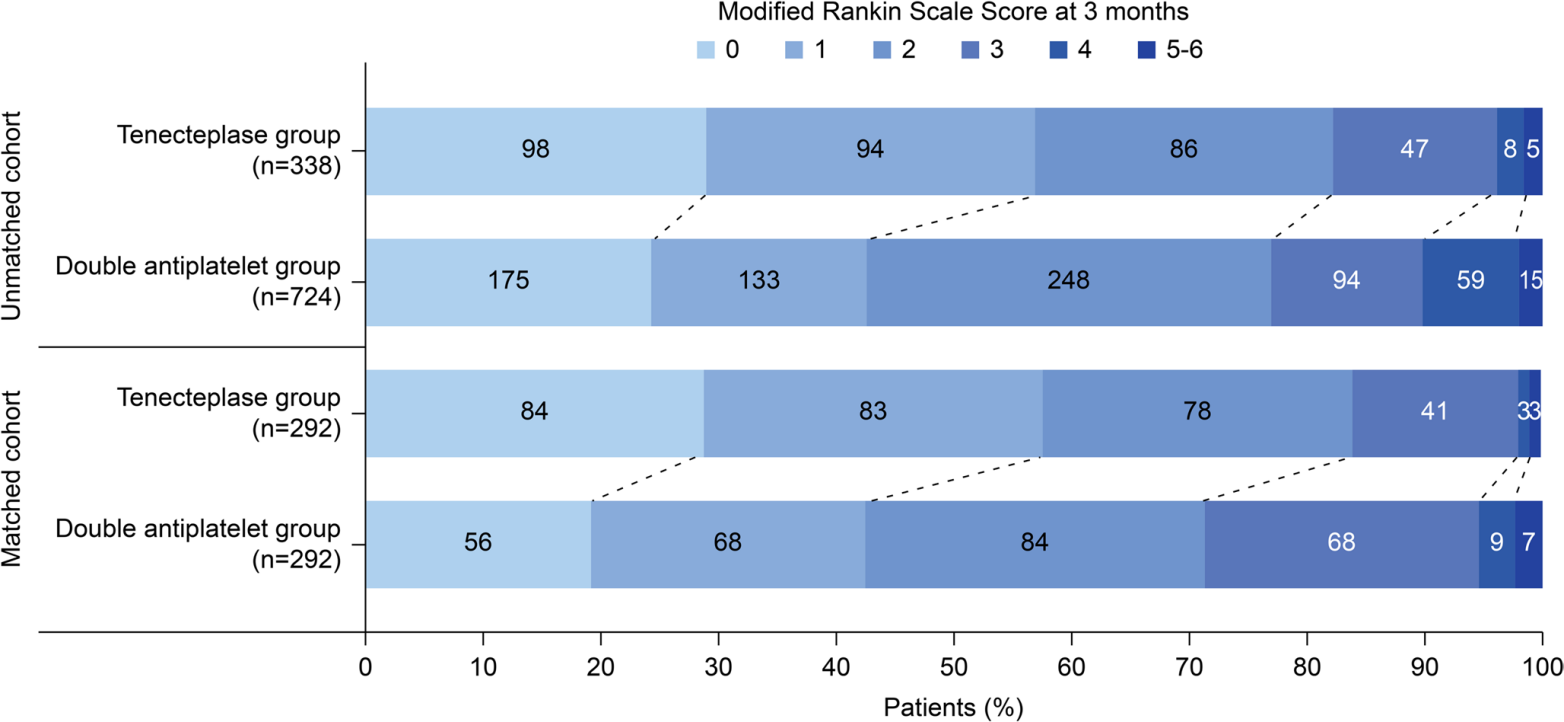
Score distribution based on the modified Rankin scale (mRS) at 3 months after stroke following intravenous thrombolysis, ranging from 0 to 6, where higher scores indicated more severe disability

 Regarding adverse events, the TNK group reported sICH in six patients (1.8%), other bleeding episodes (including gastrointestinal hemorrhage, epistaxis, gingival bleeding, subcutaneous hemorrhage, and injection site hematoma) in six patients (1.8%), and mortality in two patients (0.6%, with one attributed to pulmonary complications and one to cardiovascular events). In the DAPT group, three patients (0.4%) developed sICH, 17 patients (2.3%) experienced other bleeding events, and five patients died (0.96%, one from stroke recurrence/progression, one from pulmonary embolism, one from cardiovascular events, and two from pulmonary infection-related complications) (Table 2; Figure 2). Logistic regression analysis confirmed that the TNK group had a significantly higher risk of sICH than the DAPT group (aOR=4.756, 95% CI: 1.173–19.274; P=0.029). No significant differences were observed between the two groups in the occurrence of other bleeding events or overall mortality (both P>0.05).

**Table 2:** Study outcomes

Outcomes	Unmatched cohort				Matched cohort			
	TNK (*n* = 338)	DAPT (*n* = 724)	Multivariate binary logistic regression analysis		TNK (*n* = 292)	DAPT (*n* = 292)	Univariate binary logistic regression analysis	
			Adjusted OR (95%CI)	*P*			Unadjusted OR (95%CI)	*P*
Primary outcomes, ***n***(%)								
mRS ≤ 1 at 90 days	178(52.7)	298 (41.2)	1.587 (1.223 ~ 2.060)	< 0.001	167 (56.4)	124 (42.8)	1.733 (1.250 ~ 2.403)	< 0.001
Early neurological deterioration	50(14.8)	166 (22.9)	0.601 (0.423 ~ 0.853)	0.004	28 (9.5)	58 (19.9)	0.425 (0.262 ~ 0.689)	< 0.001
Secondary outcomes, ***n***(%)								
mRS ≤ 2 at 90 days	272(80.5)	556 (76.8)	1.241 (0.899 ~ 1.712)	0.190	245 (82.8)	208 (71.7)	1.894 (1.275 ~ 2.812)	0.002
mRS ≥ 4 at 90 days	17(5.0)	74(10.2)	0.490 (0.283 ~ 0.850)	0.011	6 (4.1)	16 (11.0)	0.346 (0.174 ~ 0.686)	0.002
Safety outcomes, ***n***(%)								
Symptomatic intracranial haemorrhage	6 (1.8)	3(0.4)	4.756 (1.173 ~ 19.274)	0.029	5 (1.7)	2(0.7)	2.509 (0.483 ~ 13.035)	0.274
Other bleeding events	6 (1.8)	17(2.3)	0.770(0.298 ~ 1.988)	0.589	6(2.0)	11(3.8)	0.532 (0.194 ~ 1.459)	0.220
Death within 90 days	2(0.6)	5(0.7)	0.845 (0.162 ~ 4.442)	0.842	2 (0.7)	3 (1.0)	0.660 (0.109 ~ 3.978)	0.650

mRS modified Rankin scale score, TNK Tenecteplase, DAPT dual antiplatelet therapy, CI confidence interval, OR odds ratio

### Outcomes in the Matched Cohort

Following PSM, the TNK group demonstrated a statistically significant enhancement compared to the DAPT group. Regarding the primary outcomes, within 90 days of stroke onset, 167 patients (56.4%) in the TNK group (n=292) achieved a favorable prognosis (mRS 0–1) compared to only 124 patients (42.8%) in the DAPT group (n=292) (OR=1.733; 95% CI: 1.250–2.403; P<0.001). Notably, the incidence of END was 9.5% (n=28) in the TNK group and 19.9% (n=58) in the DAPT group, with a significant difference between the two groups (OR=0.425; 95% CI: 0.262–0.689; P<0.001). Regarding secondary outcomes, patients in the TNK group were more prone to achieve functional independence (mRS≤2) at 90 days post-stroke (OR=1.894; 95% CI: 1.275–2.812; P=0.002).

Furthermore, the dependence rate (mRS ≥4) in the TNK group was significantly lower than that observed in the DAPT group (OR=0.346; 95% CI: 0.174–0.686; P=0.002). Regarding safety outcomes, the rate of sICH was 1.7% (n=5) versus 0.7% (n=2), the occurrence of other systemic bleeding events was 2.0% (n=6) versus 3.8% (n=11), and the mortality rate was 0.7% (n=2) versus 1.0% (n=3 cases) in the TNK and DAPT groups, respectively. Logistic regression analyses for sICH, other systemic bleeding events, and mortality did not reveal significant differences between groups (all P>0.05) (Table 2; Figure 2).

## Discussion

This study examined the efficacy and safety of TNK intravenous thrombolysis in patients with wake-up BAD presenting with DWI/FLAIR mismatch. After PSM, TNK thrombolysis significantly reduced the risk of END and improved 90-day functional outcomes compared with dual antiplatelet therapy. Higher proportions of patients in the TNK group achieved favorable mRS 0–1 and 0–2 outcomes, while the rate of poor outcomes (mRS ≥4) was significantly lower. The initially higher sICH risk in the TNK group before matching was no longer evident after adjusting for confounding factors. No significant between-group differences were observed in sICH, other bleeding events, or mortality, confirming the safety of TNK in this population.

Previous studies investigating the use of alteplase for the treatment of BAD have yielded inconsistent results. Park et al. [8] and Deguchi et al. [9] demonstrated no benefit in preventing END compared with antiplatelet therapy, whereas other studies [10, 11] indicated a reduced risk of END. A recent study reported that alteplase did not improve the overall prognosis of patients with BAD, although younger patients might achieve better outcomes without an elevated risk of hemorrhage [12].

In contrast, TNK is a genetically engineered variant of alteplase that exhibits distinct pharmacological advantages, including enhanced fibrin specificity, prolonged half-life, and diminished risk of systemic coagulopathy. [13, 14] Multiple clinical trials have consistently confirmed that for patients with AIS treated within 4.5 hours of symptom onset, TNK is non-inferior to alteplase in overall efficacy while simultaneously improving mRS-assessed functional outcomes and reducing END incidence. [15, 16] Our findings align with these prior observations on TNK and further extend this evidence base to the unique, high-risk subgroup of patients with BAD-related WUS.

Patients with wake-up BAD face two interconnected clinical challenges that complicate therapeutic decision-making. One challenge is the uncertainty surrounding the exact time of symptom onset, which has traditionally been excluded from conventional thrombolysis. The other is the inherent high risk of END, which is a key determinant of poor prognosis in BAD. Building on clinical evidence from the WAKE-UP and EXTEND trials [17, 18], which validated intravenous thrombolysis for WUS patients with DWI/FLAIR mismatch. In addition, a WAKE-UP subanalysis further supports thrombolysis in lacunar infarction, which shares a similar pathophysiology with BAD, including lipohyalinosis and microatheromatosis. These findings provide a theoretical basis for TNK use in wake-up BAD [19]. Our study provides critical guidance by demonstrating that the DWI/FLAIR mismatch criterion effectively identifies wake-up BAD patients likely to benefit from TNK thrombolysis. Unlike alteplase, the efficacy of which remains controversial in BAD, TNK offers dual advantages. These include operational convenience, as pre- and post-treatment procedures are consistent with alteplase [20, 21], and superior pharmacological properties. These attributes position TNK as a potentially preferred thrombolytic agent for wake-up BAD, supporting its integration into clinical management strategies for this subgroup and addressing the long-standing paucity of evidence-based treatments. Additionally, previous studies [22, 23] have identified 0.25 mg/kg as the optimal TNK dose for patients beyond the 4.5-hour thrombolysis window. This dosage may be applicable to wake-up BAD and provide valuable practical guidance for clinical dosing.

The pathophysiological basis of BAD involves atherosclerotic plaque formation at the ostia of penetrating arteries, leading to luminal stenosis, cerebral hypoperfusion, and eventual deep-brain infarction. END in BAD is primarily driven by progressive plaque instability, microthrombosis, or worsening hypoperfusion within 48–72 h of onset [24, 25], processes that are often exacerbated in the wake-up subtype. Wake-up BAD may be further compounded by nocturnal physiological changes, including blood pressure fluctuations, sleep apnea-induced hypoxia, and increased blood viscosity, which collectively amplify thrombogenesis and hypoperfusion in penetrating arteries that are already vulnerable. [26, 27] TNK’s enhanced fibrin specificity enables targeted lysis of microthrombi in these small vessels without inducing systemic anticoagulant effects, restoring blood flow to the ischemic penumbra and interrupting the cascade leading to END. Its extended half-life also confers sustained thrombolytic activity, addressing the dynamic and progressive thrombotic process inherent to BAD, particularly in the wake-up setting, where thrombus formation may be prolonged by nocturnal physiological stressors. [28] In contrast, the shorter half-life and lower fibrin specificity of alteplase may limit its ability to maintain effective thrombolysis against ongoing microthrombosis and hypoperfusion in BAD. This pharmacodynamic difference likely explains the inconsistent results of prior studies evaluating alteplase for this condition, including its failure to improve the overall prognosis and variable efficacy across age groups. [29] The capacity of TNK to directly target these pathophysiological cascades underpins its efficacy in reducing END and improving functional outcomes in wake-up BAD, providing a much-needed therapeutic reference for the management of this high-risk subgroup.

 Overall, this study has significant strengths. First, as one of the first studies focusing on TNK thrombolysis for wake-up BAD, it selects patients using the DWI/FLAIR mismatch criterion, effectively solves the problem of unknown symptom onset, and provides a translatable clinical diagnosis and treatment pathway. Second, the multicenter design further improves the generalizability of the research findings, and the focus on core outcome indicators such as END and 90-day mRS scores ensures that the results deliver direct therapeutic value for both patients and clinicians.

However, this study has inherent limitations that should be considered when interpreting the findings. As a multicenter retrospective analysis, variations in case selection protocols and diagnostic proficiency across centers may introduce selection bias, potentially limiting the representativeness of the study population. Data extracted from historical medical records also carries risks of missing information or non-standardized documentation, which could introduce bias into data integration and analysis. Notably, owing to the retrospective multicenter design, specific timing intervals, including the median time from awakening to imaging initiation and from awakening to treatment administration, were not uniformly documented and could not be analyzed, limiting our ability to precisely characterize treatment delays. While confounding factors were controlled for via statistical models, cluster effects inherent in multicenter data were not addressed through stratified analyses or mixed-effects models, which may impact statistical robustness. Additionally, no loading doses of aspirin or clopidogrel were administered in the dual antiplatelet therapy arm, consistent with routine clinical practice at the participating centres for wake-up BAD patients with unknown onset time. However, the absence of loading doses may have resulted in slower platelet inhibition in the DAPT group during the early post-treatment period, potentially increasing early neurological deterioration rates in this arm and biasing the comparison in favour of thrombolysis. This should be considered when interpreting the between-group differences in END. Furthermore, given the low absolute event rates for symptomatic intracranial haemorrhage (five vs. two events in the matched cohort), the study was underpowered to detect clinically meaningful between-group differences in rare safety endpoints, as reflected by the wide confidence intervals observed. The non-significant post-matching safety results should therefore be interpreted with caution and do not constitute definitive evidence of safety equivalence; future prospective studies with larger sample sizes are needed to more reliably assess the safety profile of TNK in this population.

### Conclussion

To address these limitations and build upon the current findings, future research should prioritize large-scale prospective randomized controlled trials designed to validate the efficacy and safety of TNK thrombolysis, specifically for wake-up BAD. Stratified analyses based on factors such as age, infarct location, and DWI/FLAIR mismatch characteristics are necessary to identify specific subgroups of patients who may benefit the most from TNK treatment. The application of mixed-effects models in future analyses is also recommended to account for multicenter clustering, thereby improving statistical robustness. Additionally, mechanistic studies exploring the interaction between the pharmacological properties of TNK and the unique pathophysiology of wake-up BAD may provide deeper insights into its therapeutic advantages, while long-term follow-up studies may further confirm the durability of treatment benefits and assess the risk of recurrent stroke.

## Data Availability

The datasets generated during and/or analysed during the current study are available from the corresponding author on reasonable request.

## References

[1] Duan H, Yun HJ, Geng X, Ding Y. Branch atheromatous disease and treatment. Brain Circ. 2022;8:169–71. 10.4103/bc.bc_56_22. 37181840 10.4103/bc.bc_56_22 PMC10167853

[2] Deguchi I, Takahashi S. Pathophysiology and optimal treatment of intracranial branch atheromatous disease. J Atheroscler Thromb. 2023;30:701–9. 10.5551/jat.RV22003. 37183021 10.5551/jat.RV22003 PMC10322737

[3] Sharma R, Lee K. Advances in treatments for acute ischemic stroke. BMJ. 2025;389:e076161. 10.1136/bmj-2023-076161. 40335091 10.1136/bmj-2023-076161

[4] Muir KW, Ford GA, Ford I, Wardlaw JM, McConnachie A, Greenlaw N, et al. Tenecteplase versus alteplase for acute stroke within 4·5 h of onset (attest-2): a randomised, parallel group, open-label trial. Lancet Neurol. 2024;23:1087–96. 10.1016/S1474-4422(24)00377-6. 39424558 10.1016/S1474-4422(24)00377-6

[5] Menon BK, Buck BH, Singh N, Deschaintre Y, Almekhlafi MA, Coutts SB, et al. Intravenous tenecteplase compared with alteplase for acute ischaemic stroke in Canada (act): a pragmatic, multicentre, open-label, registry-linked, randomised, controlled, non-inferiority trial. Lancet. 2022;400:161–9. 10.1016/S0140-6736(22)01054-6. 35779553 10.1016/S0140-6736(22)01054-6

[6] Peter-Derex L, Derex L. Wake-up stroke: from pathophysiology to management. Sleep Med Rev. 2019;48:101212. 10.1016/j.smrv.2019.101212. 31600679 10.1016/j.smrv.2019.101212

[7] Thomalla G, Boutitie F, Ma H, Koga M, Ringleb P, Schwamm LH, et al. Intravenous alteplase for stroke with unknown time of onset guided by advanced imaging: systematic review and meta-analysis of individual patient data. Lancet. 2020;396:1574–84. 10.1016/S0140-6736(20)32163-2. 33176180 10.1016/S0140-6736(20)32163-2 PMC7734592

[8] Park MG, Oh EH, Kim BK, Park KP. Intravenous tissue plasminogen activator in acute branch atheromatous disease: does it prevent early neurological deterioration? J Clin Neurosci. 2016;33:194–7. 10.1016/j.jocn.2016.04.011. 27452127 10.1016/j.jocn.2016.04.011

[9] Deguchi I, Hayashi T, Kato Y, Nagoya H, Ohe Y, Fukuoka T, et al. Treatment outcomes of tissue plasminogen activator infusion for branch atheromatous disease. J Stroke Cerebrovasc Dis. 2013;22:e168-72. 10.1016/j.jstrokecerebrovasdis.2012.10.012. 23246192 10.1016/j.jstrokecerebrovasdis.2012.10.012

[10] Wu X, Liu Y, Nie C, Kang Z, Wang Q, Sun D, et al. Efficacy and safety of intravenous thrombolysis on acute branch atheromatous disease: a retrospective case-control study. Front Neurol. 2020;11:581. 10.3389/fneur.2020.00581. 32733357 10.3389/fneur.2020.00581 PMC7358343

[11] Junjie C, Lijuan H, Yue L, Yang G. One case of internal capsule warning syndrome. Chin J Gen Pract. 2025;24:98–101. 10.3760/cma.j.cn114798-20240806-00667.

[12] Matsubayashi T, Fujiki S, Muramatsu R, Furuki M, Obayashi M. Efficacy of intravenous thrombolysis for the prognosis of branch atheromatous disease. Cureus. 2025;17:e81301. 10.7759/cureus.81301. 40291332 10.7759/cureus.81301 PMC12033049

[13] Wang L, Hao M, Wu N, Wu S, Fisher M, Xiong Y. Comprehensive review of tenecteplase for thrombolysis in acute ischemic stroke. J Am Heart Assoc. 2024;13:e031692. 10.1161/JAHA.123.031692. 38686848 10.1161/JAHA.123.031692 PMC11179942

[14] Miller SE, Warach SJ. Evolving thrombolytics: from alteplase to tenecteplase. Neurotherapeutics. 2023;20:664–78. 10.1007/s13311-023-01391-3. 37273127 10.1007/s13311-023-01391-3 PMC10275840

[15] Wang Y, Li S, Pan Y, Li H, Parsons MW, Campbell BCV, et al. Tenecteplase versus alteplase in acute ischaemic cerebrovascular events (TRACE-2): a phase 3, multicentre, open-label, randomised controlled, non-inferiority trial. Lancet. 2023;401:645–54. 10.1016/S0140-6736(22)02600-9. 36774935 10.1016/S0140-6736(22)02600-9

[16] Parsons MW, Yogendrakumar V, Churilov L, Garcia-Esperon C, Campbell BCV, Russell ML, et al. Tenecteplase versus alteplase for thrombolysis in patients selected by use of perfusion imaging within 4·5 h of onset of ischaemic stroke (TASTE): a multicentre, randomised, controlled, phase 3 non-inferiority trial. Lancet Neurol. 2024;23:775–86. 10.1016/S1474-4422(24)00206-0. 38880118 10.1016/S1474-4422(24)00206-0

[17] Thomalla G, Simonsen CZ, Boutitie F, Andersen G, Berthezene Y, Cheng B, et al. MRI-guided thrombolysis for stroke with unknown time of onset. N Engl J Med. 2018;379:611–22. 10.1056/NEJMoa1804355. 29766770 10.1056/NEJMoa1804355

[18] Ma H, Campbell BCV, Parsons MW, Churilov L, Levi CR, Hsu C, et al. Thrombolysis guided by perfusion imaging up to 9 hours after onset of stroke. N Engl J Med. 2019;380:1795–803. 10.1056/NEJMoa1813046. 31067369 10.1056/NEJMoa1813046

[19] Barow E, Boutitie F, Cheng B, Cho TH, Ebinger M, Endres M, et al. Functional outcome of intravenous thrombolysis in patients with lacunar infarcts in the WAKE-UP trial. JAMA Neurol. 2019;76:641–9. 10.1001/jamaneurol.2019.0351. 30907934 10.1001/jamaneurol.2019.0351 PMC6563546

[20] Rousseau JF, Weber JM, Alhanti B, Saver JL, Messé SR, Schwamm LH, et al. Short-term safety and effectiveness for Tenecteplase and alteplase in acute ischemic stroke. JAMA Netw Open. 2025;8:e250548. 10.1001/jamanetworkopen.2025.0548. 40072434 10.1001/jamanetworkopen.2025.0548 PMC11904722

[21] Hagag AM, Kormod ME, Ads ME, El-Refaay MA, Abozaid OM, Ghanem OA, et al. Tenecteplase versus alteplase in patients with acute ischemic stroke: an updated systematic review and meta-analysis. Eur J Med Res. 2025;30:726. 10.1186/s40001-025-02983-9. 40775658 10.1186/s40001-025-02983-9 PMC12333306

[22] Wang Z, Li J, Wang X, Yuan B, Li J, Ma Q. Tenecteplase for acute ischemic stroke at 4.5 to 24 hours: a meta-analysis of randomized controlled trials. Stroke. 2025;57:50–62. 10.1161/STROKEAHA.125.053256. 41078125 10.1161/STROKEAHA.125.053256 PMC12721659

[23] Waseem MH, Abideen ZU, Khan MH, Tahir MF, Khan M, Raja HAA, et al. Comparative efficacy and safety of different Tenecteplase doses with alteplase in acute ischemic stroke: a systematic review with pairwise and network meta-analysis to determine the optimal dose. Brain Behav. 2025;15:e70756. 10.1002/brb3.70756. 40827614 10.1002/brb3.70756 PMC12362178

[24] Liu Y, Wang H, Xu R, He L, Wu K, Xu Y, et al. Serum uric acid to serum creatinine ratio predicts neurological deterioration in branch atheromatous disease. Front Neurol. 2023;14:1098141. 10.3389/fneur.2023.1098141. 36741280 10.3389/fneur.2023.1098141 PMC9895829

[25] Hoshino T, Mizuno T, Arai S, Hosoya M, Ishizuka K, Higuchi E, et al. Hemostatic activation markers and early neurological deterioration in branch atheromatous disease-related stroke. J Atheroscler Thromb. 2025;32:1416–24. 10.5551/jat.65653. 40350319 10.5551/jat.65653 PMC12597478

[26] Elfil M, Eldokmak M, Baratloo A, Ahmed N, Amin HP, Koo BB. Pathophysiologic mechanisms, neuroimaging and treatment in wake-up stroke. CNS Spectr. 2020;25:460–7. 10.1017/S1092852919001354. 31511119 10.1017/S1092852919001354

[27] Barreto PR, Diniz DLO, Lopes JP, Barroso MC, Daniele TMDC, de Bruin PFC, et al. Obstructive sleep apnea and wake-up stroke – a 12 months prospective longitudinal study. J Stroke Cerebrovasc Dis. 2020;29:104564. 10.1016/j.jstrokecerebrovasdis.2019.104564. 31866199 10.1016/j.jstrokecerebrovasdis.2019.104564

[28] Ahmed HK, Logallo N, Thomassen L, Novotny V, Mathisen SM, Kurz MW. Clinical outcomes and safety profile of Tenecteplase in wake-up stroke. Acta Neurol Scand. 2020;142:475–9. 10.1111/ane.13296. 32511749 10.1111/ane.13296

[29] Meng X, Li S, Dai H, Lu G, Wang W, Che F, et al. Tenecteplase vs alteplase for patients with acute ischemic stroke: the original randomized clinical trial. JAMA. 2024;332:1437–45. 10.1001/jama.2024.14721. 39264623 10.1001/jama.2024.14721 PMC11393753

